# The biobehavioral Women’s Health CoOp in Pretoria, South Africa: study protocol for a cluster-randomized design

**DOI:** 10.1186/1471-2458-14-1074

**Published:** 2014-10-15

**Authors:** Wendee M Wechsberg, William A Zule, Jacqueline Ndirangu, Tracy L Kline, Nathaniel F Rodman, Irene A Doherty, Scott P Novak, Charles M van der Horst

**Affiliations:** RTI International, 3040 E. Cornwallis Road, PO Box 12194, Research Triangle Park, NC 27709-2194 USA; RTI International, 258 Burgers Park Lane, Pretoria, South Africa; UNC Chapel Hill School of Medicine, Manning Drive, Chapel Hill, NC 27599-7030 USA

**Keywords:** Vulnerable women, HIV, South Africa, Combined biobehavioral intervention, Women’s Health CoOp, Seek, test, treat and retain paradigm, Cluster-randomized design

## Abstract

**Background:**

South Africa has 6.4 million adults over the age of 15 living with HIV. Gender inequality issues continue to drive the HIV epidemic in South Africa, where Black African women bear the greatest HIV burden. Limited access to services; little capacity to negotiate sex and condom use; and other legal, social, and economic inequities make women highly vulnerable to HIV infection. Behavioral interventions have been shown to decrease risk behaviors, but they have been less successful in reducing HIV incidence. Conversely, biomedical prevention strategies have proven to be successful in reducing HIV incidence, but require behavioral interventions to increase uptake and adherence. Consequently, there is a need for integrated approaches that combine biomedical and behavioral interventions. Effective combination prevention efforts should comprise biomedical, behavioral, and structural programming proven in randomized trials that focuses on the driving forces and key populations at higher risk of HIV infection and transmission.

**Methods/Design:**

This prospective, geographically clustered randomized field experiment is enrolling participants into two arms: a control arm that receives standard HIV testing and referral for treatment; and an intervention arm that receives an evidence-based, woman-focused behavioral intervention that emphasizes risk reduction and retention, the Women’s Health CoOp. We divided the city of Pretoria into 14 mutually exclusive geographic zones and randomized these zones into either the control arm or the intervention arm. Outreach workers are recruiting drug-using women from each zone. At baseline, eligible participants complete a questionnaire and biological testing for HIV, recent drug use, and pregnancy. Follow-up interviews are completed at 6 and 12 months.

**Discussion:**

The biobehavioral intervention in this study merges an efficacious behavioral HIV prevention intervention for women with biomedical prevention through HIV treatment as prevention using a Seek, Test, Treat and Retain strategy. This combination biobehavioral intervention is designed to (1) improve the quality of life and reduce HIV infectiousness among women who are HIV positive, and (2) reduce HIV risk behaviors among women regardless of their HIV status. If efficacious, this intervention could help control the HIV epidemic in South Africa.

**Trial registration:**

Trial registration no: NCT01497405.

## Background

South Africa has the highest number of people living with HIV in the world. HIV prevalence increased in South Africa from 10.6% in 2008 to 12.2% in 2012 [1]. This increase likely reflects more accurate estimates arising from the widespread testing campaigns and a reduction in HIV-related mortality as a result of the large-scale expansion of antiretroviral therapy (ART) programs [2] coupled with continued HIV incidence. Although the epidemic is generalized, a number of concentrated subepidemics exist among specific groups that have HIV prevalence above the national average [3], including young Black African women and high-risk alcohol and recreational drug users. A survey by the South African Human Science Research Council (HSRC) reported that although the epidemic has stabilized, Black African women are still the most affected. HIV prevalence among Black African women aged 15 to 49 is 23.2%, whereas prevalence among their male counterparts is 18.8% [4]. The HSRC survey also found that among adults aged 15 to 49, the number of new infections was 1.7 times as high among females as among males. Moreover, among females aged 15 to 24 the HIV incidence rate was over four times higher than among males in this same age group.

Although 37% of men and women between the ages of 15 and 49 have been tested for HIV, testing rates are much lower among key populations, such as female sex workers or women who use alcohol or other drugs (AOD). This is problematic because HIV prevalence among female sex workers exceeds 50% in some areas [5], 65% in South Africa, and HIV prevalence among women who use AOD but are not sex workers is often greater than 30% [6]. Despite the high prevalence of HIV, only 60% of female sex workers are reached by current HIV prevention programs (e.g., HIV testing, condom distribution) [7]. This relatively low rate of HIV testing in this key population represents a missed public health opportunity [8].

Economically disadvantaged women, many of whom use AOD, are often responsible for supporting themselves and their extended families. Under these circumstances, they may resort to sex work and engage in high rates of unprotected sex with multiple partners [9]. A majority of these partners also have concurrent partnerships, which further contributes to the spread of HIV in South Africa [10]. A study in Ghana found that 84% of HIV infections among males aged 15 to 59 could be attributed to transactional sex [11]. In simulation models, the presence of sex workers led to a substantially larger heterosexual HIV epidemic. Taken together, the findings from the epidemiological and modeling studies suggest that interventions to reduce risk for sex workers could slow the epidemic [12].

Biomedical interventions, such as increasing the percentage of people living with HIV that receive ART, have been associated with reducing HIV transmission [13]. One meta-analysis of 11 cohort studies of discordant couples estimated that ART in the index case resulted in a 92% reduction in HIV incidence [14]. Other studies that examined the effect of HIV treatment at the community level reported associations between reductions in community viral load and reductions in HIV incidence [15, 16]. These findings suggest that widespread use of ART can reduce HIV transmission at the community level.

ART and other biomedical interventions require adherence to the medications to be effective. Without adherence, HIV antiretroviral (ARV) resistance mutations can occur, leading to loss of viral suppression with risk to the patients and their sexual partners. Substance abuse is a critical barrier to ART adherence [17]. Widespread access to ART may lead to riskcompensation, i.e., an increase in risky behavior caused by actual or perceived lower risk. Risk compensation occurs when people feel that they are no longer at risk of HIV infection or transmission [18]. The combined effect of incomplete viral suppression and risk compensation could potentially further increase transmission to partners. Consequently, it is important for biomedical HIV interventions to continue to emphasize behavioral prevention, particularly condom use even after initiating ART [19].

While biomedical intervention strategies reduce the probability of HIV acquisition and transmission, behavioral interventions promote reductions in risk behaviors, adherence to medications and clinic visits, and condom use; however, few are specifically gender interventions focused on women’s issues. In addition, there is growing recognition that combination interventions with biomedical and evidence-based behavioral strategies coupled with structural change components—such as increased access to ART and substance abuse treatment—are more effective in reducing HIV transmission than any of these strategies alone [20–22]. Prevention packages that combine effective woman-focused interventions should be implemented and scaled up to achieve a measurable reduction in population-level HIV prevention [23].

## Methods/Design

### Objectives

The aim of this study is to assess the relative impact of adding the Women’s Health CoOp (WHC) to standard Test, Treat, and Retain (TTR) practices on the numbers of HIV-positive, AOD-using women who receive medical evaluations (e.g., CD4, viral load), initiate treatment, remain in treatment, and have suppressed viral load. It will also assess the relative impact of adding the WHC to TTR practices on reductions in risk behaviors (e.g., AOD use, not using condoms, victimization) among all women, regardless of their HIV status.

### Study design

**Figure 1:**
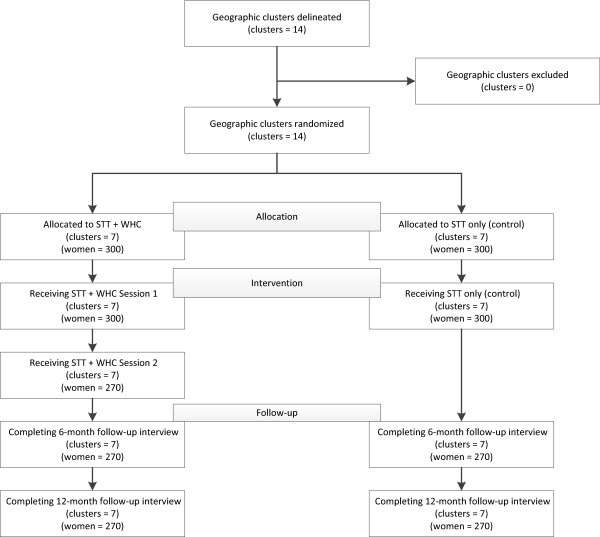
**Anticipated recruitment, intervention participation, follow-up, and allocation of clusters.**

This study is a prospective, geographically clustered randomized two-group field experiment. It is being conducted in 14 geographic clusters in Pretoria, South Africa (Figure 1). We chose the cluster randomized design to minimize contamination between study arms. The study will assess the efficacy of a combined HIV/AIDS prevention/intervention and enhanced strategy targeting AOD-using women for ART initiation, adherence, retention, and reduced risk behaviors compared with standard TTR practices in South Africa. Recruitment started in 2012 and will end in 2014. Follow-up interviews conducted at 6 and 12 months post-enrollment will continue through 2015.

### Setting

We divided the entire city of Pretoria—which includes a central business district, other commercial areas, and residential areas such as a number of formal and informal townships and settlements—into 14 mutually exclusive “zones.” We delineated the zones according to natural boundaries based on consultation with outreach workers who were knowledgeable about Pretoria.

### Randomization

We created seven pairs of zones that were matched on the basis of socioeconomic conditions and estimates of the number of sex workers and women who use AOD residing in them. These estimates were based on inputs from our outreach workers who had worked on one of our prior studies that reached drug-using, at-risk women and sex workers in the Pretoria area [23–25].

We randomized zones within each pair to the control arm or the intervention arm. Each participant’s study arm is determined by the zone in which she was recruited.

### Study population

Participant eligibility criteria include the following: (1) Female; (2) Black African; (3) Aged 15 or older (if under 18, must be able to demonstrate the criteria for tacit emancipation according to South African law); (4) Report using at least one of the following drugs weekly during the past 90 days—alcohol, marijuana (dagga), methamphetamine (tik), methaqualone (Mandrax), cocaine (crack and/or powder), heroin (Thai White), inhalants (glue and benzene), MDMA (ecstasy), LSD, methcathinone (Kat/cat), or marijuana and heroin mixed (Nyaope); (5) Report having unprotected vaginal sex with a male partner in the past 6 months; (6) Speaks English, Sesotho, Tswana, or Zulu; (7) Consent to HIV rapid testing and drug testing; (8) Provide written and verbal assent/consent to participate; and (9) Provide verifiable locator information (e.g., house, street corner, vacant lot) for the Pretoria area and plan to stay in the area for the next 12 months. Exclusion criteria include the following: (1) Male; and (2) Self-identify as other than Black African.

### Ethical approval

This study is approved by the South African Medical Association Research Ethics Committee (SAMAREC), Tshwane Research Committee (TRC), and the RTI International Committee for the Protection of Human Subjects. All participants are required to give their informed consent prior to any study procedure. We obtained a waiver for parental consent for participants under the age of consent, which in South Africa is 18 years. Adolescents aged 12 or older can legally consent to HIV testing and medical treatment in South Africa. Also, minors having children, owning a business, or having an occupation that brings in a salary and residence outside the parental home are recognized as emancipated.

When we collect locator information, we explain to the women that we will be coming to look for them for their follow-up visits. Outreach workers are trained to describe the project as a health project, and they are also trained not to disclose any confidential information. All of our staff complete ethics training annually.

### Data and Safety Monitoring Board

The study has established a Data and Safety Monitoring Board (DSMB) comprising expert physicians in infectious diseases, epidemiology, HIV treatment in South Africa, and bioethics. One physician resides in South Africa and treats patients, one is a bioethicist, and the chair is also an infectious disease physician and epidemiologist. The DSMB receives progress reports and meets biannually with the investigators and receives notification and information regarding the circumstances surrounding any participant deaths.

As specified in our Data and Safety Monitoring Plan (DSMP), Severe Adverse Events (SAEs) are reported to the Principal Investigator within 24 hours and to the DSMB, the funding agency and the Institutional Review Board within 48 hours, with appropriate action taken immediately.

### Study procedures

#### Recruitment

Outreach workers actively market the study in each of the zones to recruit participants. The outreach model evolved from a long history of outreach work that started in North Carolina [26], which was later modified for use in Pretoria and Cape Town [27, 28]. We trained the outreach workers on how to market the study in the zones and how to find female sex workers and other vulnerable women who use AOD. The training also includes activities to ensure that outreach workers are aware of safety measures and how to avoid potentially dangerous situations they may encounter. Outreach workers conduct outreach in a wide range of settings that vulnerable women frequent, including daily-rate hotels, taverns, pool halls, brothels, bushes and thickets, and on street corners. Outreach workers also market the study to “big mammas” (madams) and pimps who often serve as gatekeepers for sex workers.

To enroll in the study, eligible participants travel to the field site where the baseline appointment takes place. If participants are not able to come to the field site, interviews may be conducted in a private setting at or near the participant’s residence or place of work. In these instances staff follow a protocol to ensure confidentiality. All outreach workers are familiar with the city of Pretoria and fluent in the local languages, and they are trained on how to approach, screen, and recruit marginalized and stigmatized vulnerable women.

#### Modifications to the protocol

##### Recruitment

Originally, we proposed to recruit the sample by placing mobile units in selected “hotspots” for both study arms to serve as recruitment venues. The hotspots are well-known areas in the community. In the areas randomized to the treatment arm, we planned to enhance recruitment with active outreach to bring women to the mobile unit. However, when funding for the study was awarded, the mobile units were no longer available. This required us to divide the entire city of Pretoria into zones. Consequently, outreach workers market the study and recruit women as described above and they are brought to a field site in the center of the city.

##### Eligibility criteria

The original eligibility criteria included the use of two drugs (one of which could be alcohol) at least weekly. These criteria excluded a large proportion of sex workers and other high-risk women, many of whom were HIV positive. Therefore, we changed eligibility criteria to the use of alcohol or another drug at least weekly.

##### Intake process

In most instances, staff conduct the intake process (i.e., informed consent, baseline interview, biological testing) at the field site. However, in instances in which a participant is not able to come into the field site, project staff conduct the intake in a private setting in the field.

##### Intervention case management

We initially planned to conduct case management weekly using cell phones in most instances. However, many women did not have cell phones, so staff had to conduct case management face to face in many instances, which was more labor intensive. To offset this burden on staff, we reduced the frequency of case management from weekly to monthly.

##### Sample size

We reduced the sample size from 1050 to 600 minimum. The section on sample size includes a description of the rationale for this change.

##### Changes in incentives

We increased the incentives for baseline interviews from R70 to R100 ($1 US equals approximately 10 Rand). We also increased the incentives for 6-month follow-up interviews from R100 to R150 and for 12-month interviews from R150 to R200.

##### Miscellaneous changes

Local HIV clinics required CD4 cell count tests before starting patients on ART. However, there was a backlog for CD4 testing, which resulted in extensive delays in starting ART. To remove this barrier, we began providing onsite CD4 testing. Some women who were on ART reported not having a secure place to store their ARVs. To alleviate this problem, we began storing ARVs for women who did not have a place to store them. We also began conducting some follow-up interviews in private settings in the field if a participant could not come to the field site. We began collecting dried bloodspots for future HIV viral load testing. This was necessary because many HIV clinics were unable to provide HIV viral load test results, and there was no source of viral information for women who were not in HIV treatment (see Table 1 for a list of modifications by date).

**Table 1:** **Modifications to the protocol**

Date	Amendment
6/2012	Started completing full intake process (i.e., consent, biological testing, and baseline interview) in the field in addition to the field site.
6/2012	Dropped recruitment through mobile units and added recruitment through outreach workers with most activities conducted at fixed field site. [Note: mobile units were never used to recruit any participants for the study]
6/2012	Reduced frequency of case management from weekly to monthly.
11/2012	Changed eligibility criteria from weekly use of 2 or more drugs (including alcohol) to weekly use of 1 or more drugs (including alcohol).
12/2012	Provided space onsite to store ARVs for participants who did not have a place to store them.
10/2013	Reduced proposed sample from 1050 to 600 minimum.
12/2013	Added onsite CD4 testing.
1/2014	Started completing some follow-up interviews in the field when participants were unable to return to the field site.
5/2014	Increased incentives for baseline interview from R70 to R100; for 6-month follow-up interviews from R100 to R150; and for 12-month follow-up interviews from R150 to R200. [Note: Rand to Dollar conversion rate is approximately R10 = US $1]
5/2014	Began collecting and storing dried bloodspots for future HIV viral load testing.

#### Study activities by arm

##### Control arm

Participants in the control arm receive the “standard HIV counseling and testing” protocol performed in South Africa. At intake, participants complete an interviewer-administered baseline assessment [27] using Computer-Assisted Personal Interviewing (CAPI) technology. Project staff collect biological specimens and test them for evidence of recent drug use, for pregnancy, and for HIV. They also provide HIV pretest and posttest counseling. If a participant tests positive for HIV, her posttest counseling is followed by CD4 testing, which is done onsite immediately afterward using a PIMA™ analyzer (http://pimatest.com/en/home.html). In accordance with medical practice in South Africa, after receiving the CD4 test results, the participant is referred to the appropriate clinic for further medical evaluation and clinical staging that determines whether she is eligible to initiate ART (CD4<350). If the participant initiates ART, she receives adherence counseling through the health department clinic and a full battery of tests (blood count, liver function, and kidney tests) in preparation for ART initiation. The participant also receives CD4 testing at 6- and 12-month follow-up visits. HIV-positive people who are not eligible to receive ART because their CD4 cell count is >350 are referred to a “wellness program.” The local health department clinics operate the wellness programs, which provide access to support groups, ongoing adherence counseling, and information about reproductive health, nutrition, and positive living. When appropriate, the project field staff also provides referrals for pregnancy services, tuberculosis testing, substance abuse rehabilitation, and professional counseling for gender-based violence. Staff members also provide simple meals and coordinate childcare for women in the study during their appointments. They also arrange transportation to the field site for the appointments.

#### Intervention arm

Women in the intervention arm participate in all of the activities described above for the control arm. In addition, they participate in two individual, woman-focused HIV prevention intervention sessions. These sessions aim to increase knowledge, skills, and motivation to make behavioral changes related to AOD use, sexual risk, and violence and victimization. These sessions take place at the field site and are facilitated by an experienced female interventionist who is a multilingual peer leader from the community and who also is sensitive to helping women develop empowerment strategies with a personalized plan after the intervention session.

The WHC is an evidence-based, woman-focused, behavioral intervention that reaches high-risk, hard-to-reach, vulnerable women. It addresses the intersecting risks of drug use, sex behaviors, violence and victimization, with the primary goal of increasing skills and knowledge. The WHC has been adapted from previous iterations in North Carolina, Rhode Island, Massachusetts, Russia, the Republic of Georgia, and South Africa. It is based on social cognitive theory and empowerment theory [29]. Two intervention sessions are held approximately 3 to 5 days apart. Each session lasts 30 to 45 minutes. Key elements of the sessions educate participants about the risks of AOD use and how AOD use and sex risk relate to HIV for women. The sessions also teach risk-reduction methods such as proper condom use, sexual negotiation, and violence prevention strategies. Women also role-play and rehearse how to use male and female condoms correctly, as well as condom negotiation. During the sessions, the women create personalized action plans in which they develop a concrete plan to reduce risk. At the end of the second session, the participant completes the personalized plan, which she takes home as a guideline for her set goals. The staff conducts case management face to face or by cell phone at least monthly to follow up and support the participant in her risk-reduction and action plan to improve her life. For women with AOD use problems, the interventionists work closely with drug rehabilitation facilities to secure treatment slots.

Because this adaptation of the WHC combines TTR activities with a specific focus on linking women to care if they are newly or previously diagnosed with HIV before enrollment, we named this combined biobehavioral intervention the Women’s Health CoOp Plus (WHC+).

#### Outcome measures

The primary outcome is self-reported male or female condom use during the participant’s most recent episode of vaginal or anal intercourse. Other outcomes include frequency of condom use, frequency and quantity of alcohol use, urine drug screen results for illicit substance use, and frequency of violent victimization (physical or sexual). Additional outcomes for women living with HIV include receiving HIV treatment, ART adherence, and CD4 counts. Analyses for HIV-RNA viral load on stored dried blood spots at follow-up may occur at a later date.

Alcohol use is measured biologically with a breathalyzer test and through self-report. A biological urine drug screening test determines recent use of a panel of substances, including cannabis/marijuana, opioids, cocaine, methamphetamine, and Mandrax (methaqualone). Victimization includes self-report of being beaten, attacked with a weapon, or forced to have sex during the previous 90 days. We assess if women living with HIV received treatment and took ART both biologically and by self-report. Self-report for ART refers to being prescribed ART, taking ART if prescribed, and missing any doses in the past 30 days. We also examine measures of adherence included in the U.S. National Institute on Drug Abuse’s harmonization initiative [30]. To help interpret these data, we also collect self-reports of barriers to ART use.

#### Staff training and quality assurance

In addition to the outreach training, field staff receive hands-on training with the Principal Investigator and with a guided field manual. They also receive ongoing training and support for the various expressions of emotional distress and crisis management. Interventions and interviews are audiotaped for quality assurance and all files are checked by a staff member and then checked again and signed off by the Field Project Manager. Training for staff also covers confidentiality and ethics. All staff members must sign a confidentiality agreement. They are also educated on their responsibilities for securing the consent forms and screeners and on the confidentiality requirements of the project.

#### Data collection, data management, and quality assurance

To protect confidentiality, the study assigns each participant a unique study identification code (ID). This ID is the only link between the behavioral and biological data and the identifying information collected for locating participants for their follow-up interviews. Locator information is stored separately from other data in a double-locked file cabinet that is in a locked room with restricted access.

Data collection for this study is conducted by highly trained local staff that develop a rapport with the study participants to engender trust and elicit the most accurate data possible. Highly trained local interviewers administer questionnaires using CAPI software that has been programmed to check that responses to each question are within the valid range, that responses to different questions are logically consistent, and to skip questions that are not applicable. Biological testing information is keyed into the same software system and rechecked for accuracy. Data are transmitted each day from the field site to secure servers at RTI’s headquarters in the United States. The US-based Data Manager reviews additional automated quality control checks that the software generates each day. The Principal Investigator, other members of the research team, and the field staff also receive these reports. If any critical inconsistencies are noted, the Data Manager contacts the Field Project Manager who works with the field staff member who collected or entered the data to resolve the inconsistency.

#### Sample size and power

The sample size was lowered to 600 from 1050 as originally planned. The original sample size was calculated on the basis of an anticipated HIV prevalence of 30%; however, HIV prevalence in the sample is 55%. Accordingly, the smaller sample (n = 600) will allow for the identification of a clinically meaningful effect with sufficient statistical power, while accounting for logistical difficulties, including a citywide study, limited structural resources, increased costs, and other factors outside the control of the study. We assume 14 clusters, approximately 30 observations per cluster and 20% attrition, yielding a final sample size of about 480 respondents at 12-month follow-up. This allows us a medium to strong likelihood of detecting an effect of the intervention assuming a strong effect (based on Cohen’s beta of 0.6). This would translate to an approximately 10% to 15% difference in the outcomes for any behavioral outcome (e.g., reductions in AOD use, number of instances of unprotected sex). We would not be able to detect mild to weak effects in analyses that consider the mediation of the effect of the intervention through some cognitive outcome, such as intentions/self-efficacy/knowledge/gender power. The reduction in power also greatly reduces the likelihood of testing whether the intervention yielded modification effects across the levels of some covariate and mediational effects as well. However, we will examine the distribution of the variables and reestimate power given the final sample size and variance estimates. No outcome or mediator/moderator analyses will be conducted unless there is strong power (e.g., Beta greater than .8, for a 2-tailed test and p = .05).

#### Analysis

We will first conduct descriptive statistical analysis of baseline variables and clinical characteristics of the two intervention arms to examine the extent, if any, of baseline or clinical imbalances between the two arms using contingency table analyses (Mantel-Haenszel for categorical and analysis of variance for continuous variables). The analysis will center around estimating the impact of the intervention on each of the primary study outcomes, observed at baseline and 6- and 12-month follow-up visits, using all available cases consistent with an intent-to-treat method. In addition to evaluating changes in each outcome by intervention arm at 6-month follow-up, the analysis will examine whether the changes are sustained or decay at 12-month follow-up. Given the longitudinal nature of the data, our primary analytic strategy will apply a generalized estimating equation (GEE) modeling approach to account for the multiple dependencies of repeated measures collected on the same individuals over time, and the clustering of the outcomes by community. Because the communities may range from 5 to 100 cases, the generalized linear/nonlinear mixed model (e.g., hierarchical linear modeling or multilevel modeling with random effects) would likely face problems with convergence given the heterogeneity in the variation between communities. Therefore, we opt for a statistical strategy that treats this level of clustering as a nuisance parameter, and thereby estimating the population-averaged effects of the outcomes for subjects sharing the same profile of individual and community-level characteristics. For outcomes with binomial or count distributions, the logit link will be specified to obtain effect estimates of the magnitude of differences between arms within each follow-up visit. For continuously distributed outcomes, an identity link function will be specified. We expect that we will estimate the working correlation matrix at the temporal level using a first-order autoregressive structure (AR-1) and an exchangeable correlation matrix at the community-level, thereby allowing the subjects within each cluster to be constrained to equality but freeing the estimates between clusters.

In addition to the main outcome analyses, we will examine the mechanism of change via standard mediational models. For this approach, we will lag the timepoints such that the baseline will be the most exogenous, then the midpoint 6-month observation will be the mediator and the 12-month observation will serve as the distal outcome. We can also estimate a mediational model with two timepoints, estimating the distal outcome at 6 months along with the concurrent mediator. These models will be fit using standard structural equation modeling software packages (e.g., Mplus), which can adjust the standard errors via bootstrapping to reduce bias and also remove exogenous measurement error. These models can be estimated using a mixture of distributions, such as a categorical mediator or outcome in concert with a continuously distributed mediator or outcome. The mediational analyses will address the extent to which the intervention worked and estimate the direct and indirect mechanisms. However, moderational analyses will address the extent to which the intervention worked for specific subgroups, such as women who are HIV positive, women in a relationship, and women with a criminal history. For these analyses, interaction terms will be created by multiplying the effect of the intervention by the moderator variable of interest.

## Discussion

Behavioral and biomedical interventions typically involve separate research silos. This project bridges these fields with a biobehavioral intervention that aims to reduce risk behavior and victimization and improve HIV-related clinical outcomes. This blend of biological and behavioral outcomes, which we assess using biological tests and self-reports, will help to inform how to further enhance the use of women-centered interventions in clinical settings.

This study comprises four important innovations: (1) a citywide outreach component to increase the number of high-risk women reached; (2) an intervention case management component to increase HIV treatment initiation and retention in women who test positive for HIV; (3) evidence-based, gender-focused behavioral counseling to reduce risk behavior and increase ART adherence among women who are HIV positive; and (4) evidence-based risk-reduction counseling for women who are HIV negative. This last innovation is especially important in a country like South Africa with high HIV prevalence, where even people who exhibit a low level of risk behavior are nonetheless likely to be exposed to HIV.

The urine drug screens, HIV viral load tests, and CD4 cell count tests strengthen the scientific rigor of the study. Using biologically confirmed outcomes in addition to self-report will increase confidence in the findings regarding intervention efficacy. The new protocol for the WHC in this study has enhanced the intervention by integrating biomedical elements into the empowerment intervention to produce a combination of biobehavioral outcomes. This combination of outcomes will increase the efficacy of the intervention and improve our understanding of women’s vulnerability.

Studies show that HIV testing alone has little impact on risk behavior among people who test negative for HIV [31, 32]. Combining the WHC with the current test, treat, and retain activities can potentially increase the efficiency of the TTR strategy substantially. The combination prevention approach ensures that women who are HIV negative receive the WHC intervention, which has been shown to reduce HIV risk behavior [25, 28]. The combination approach also links women who are HIV positive to treatment, and supports their retention in treatment through case management. If this combination approach demonstrates efficacy, the WHC component is already packaged and ready for rapid implementation in real-world settings, which increases the likelihood that it will have a significant public health impact in the foreseeable future.

### Trial Status

Recruiting.

## Abbreviations

AOD: Alcohol or other drug
ART: Antiretroviral therapy
AR-1: First-order autoregressive structure
CAPI: Computer-Assisted Personal Interviewing
DSMB: Data and Safety Monitoring Board
DSMP: Data and Safety Monitoring Plan
GEE: Generalized estimating equation
HSRC: South African Human Science Research Council
SAEs: Severe Adverse Events
SAMAREC: South African Medical Association Research Ethics Committee
STTR: Seek, test, treat, and retain
TRC: Tshwane Research Committee
TTR: Standard test, treat, and retain
WHC: Women’s Health CoOp
WHC+: Women’s Health CoOp Plus.
